# Relationship between coronary high-intensity plaques on T1-weighted imaging by cardiovascular magnetic resonance and vulnerable plaque features by near-infrared spectroscopy and intravascular ultrasound: a prospective cohort study

**DOI:** 10.1186/s12968-023-00916-1

**Published:** 2023-01-30

**Authors:** Tatsuya Fukase, Tomotaka Dohi, Shinichiro Fujimoto, Ryota Nishio, Yui O. Nozaki, Ayako Kudo, Mitsuhiro Takeuchi, Norihito Takahashi, Yuichi Chikata, Hirohisa Endo, Yuko O. Kawaguchi, Shinichiro Doi, Hiroki Nishiyama, Makoto Hiki, Iwao Okai, Hiroshi Iwata, Takayuki Yokoyama, Shinya Okazaki, Katsumi Miyauchi, Hiroyuki Daida, Debiao Li, Yibin Xie, Tohru Minamino

**Affiliations:** 1 Department of Cardiovascular Biology and Medicine, Juntendo University Graduate School of Medicine, 2-1-1 Hongo, Bunkyo-Ku, Tokyo, 113-8421 Japan; 2 Department of Radiological Technology, Faculty of Health Science, Juntendo University, 2-1-1 Hongo, Bunkyo-Ku, Tokyo, 113-8421 Japan; 3 Cedars-Sinai Medical Center, Biomedical Imaging Research Institute, Los Angeles, CA USA; 4 Japan Agency for Medical Research and Development-Core Research for Evolutionary Medical Science and Technology (AMED-CREST), Japan Agency for Medical Research and Development, 1-7-1 Otemachi, Chiyoda-Ku, Tokyo, 100-0004 Japan

**Keywords:** High-intensity plaque, Cardiovascular magnetic resonance, Near-infrared spectroscopy, Intravascular ultrasound, Vulnerable plaque, Stable coronary artery disease

## Abstract

**Background:**

This study aimed to compare the coronary plaque characterization by cardiovascular magnetic resonance (CMR) and near-infrared spectroscopy (NIRS)-intravascular ultrasound (IVUS) (NIRS-IVUS), and to determine whether pre–percutaneous coronary intervention (PCI) evaluation using CMR identifies high-intensity plaques (HIPs) at risk of peri-procedural myocardial infarction (pMI). Although there is little evidence in comparison with NIRS-IVUS findings, which have recently been shown to identify vulnerable plaques, we inferred that CMR-derived HIPs would be associated with vulnerable plaque features identified on NIRS-IVUS.

**Methods:**

52 patients with stable coronary artery disease who underwent CMR with non-contrast T1-weighted imaging and PCI using NIRS-IVUS were studied. HIP was defined as a signal intensity of the coronary plaque-to-myocardial signal intensity ratio (PMR) ≥ 1.4, which was measured from the data of CMR images. We evaluated whether HIPs were associated with the NIRS-derived maximum 4-mm lipid-core burden index (maxLCBI_4mm_) and plaque morphology on IVUS, and assessed the incidence and predictor of pMI defined by the current Universal Definition using high-sensitive cardiac troponin-T.

**Results:**

Of 62 lesions, HIPs were observed in 30 lesions (48%). The HIP group had a significantly higher remodeling index, plaque burden, and proportion of echo-lucent plaque and maxLCBI_4mm_ ≥ 400 (known as large lipid-rich plaque [LRP]) than the non-HIP group. The correlation between the maxLCBI_4mm_ and PMR was significantly positive (r = 0.51). In multivariable logistic regression analysis for prediction of HIP, NIRS-derived large LRP (odds ratio [OR] = 5.41; 95% confidence intervals [CIs] 1.65–17.8, *p* = 0.005) and IVUS-derived echo-lucent plaque (OR = 5.12; 95% CIs 1.11–23.6, *p* = 0.036) were strong independent predictors. Furthermore, pMI occurred in 14 of 30 lesions (47%) with HIP, compared to only 5 of 32 lesions (16%) without HIP (*p* = 0.005). In multivariable logistic regression analysis for prediction of incidence of pMI, CMR-derived HIP (OR = 5.68; 95% CIs 1.53–21.1, *p* = 0.009) was a strong independent predictor, but not NIRS-derived large LRP and IVUS-derived echo-lucent plaque.

**Conclusions:**

There is an important relationship between CMR-derived HIP and NIRS-derived large LRP. We also confirmed that non-contrast T1-weighted CMR imaging is useful for characterization of vulnerable plaque features as well as for pre-PCI risk stratification.

*Trial registration* The ethics committee of Juntendo Clinical Research and Trial Center approved this study on January 26, 2021 (Reference Number 20-313).

**Supplementary Information:**

The online version contains supplementary material available at 10.1186/s12968-023-00916-1.

## Background

The concept of vulnerable plaque was initially proposed in 1989 [1], and is defined as an atheromatous plaque at risk of developing cardiovascular events, such as acute coronary syndrome and sudden cardiac death, following future thrombus formation [2–4]. The gold standard for vulnerable plaque detection is invasive coronary imaging, such as intravascular ultrasound (IVUS) or optical coherence tomography. Among these modalities, near-infrared spectroscopy (NIRS)-IVUS (NIRS-IVUS) is a new intravascular diagnostic imaging system that can simultaneously evaluate the plaque morphology and the presence of lipid-core plaques, and may be able to identify vulnerable plaques [5, 6], and is useful for the prediction of peri-procedural myocardial infarction (pMI) [7].

Non-invasive imaging techniques are easy to perform in clinical practice as alternatives to these invasive imaging techniques. Above all, the coronary atherosclerosis T1-weighted characterization (CATCH) technique in cardiovascular magnetic resonance (CMR) is able to acquire bright-blood reference images and dark-blood T1-weighted images quickly and simultaneously, without contrast and with high spatial resolution, and can identify vulnerable plaques as high-intensity plaques (HIPs) [8]. Some previous studies have shown that the presence of HIPs on CMR was associated with the findings of plaque vulnerability detected using invasive imaging techniques [8–10], but not with NIRS findings. Therefore, this study aimed to clarify the relationship between HIPs on non-contrast T1-weighted imaging using CMR and clinically vulnerable plaque features using NIRS-IVUS, and to determine whether pre-percutaneous coronary intervention (PCI) evaluation using CMR identifies HIPs at risk of pMI.

## Methods

### Ethics statements

This study was approved by the ethics committee of our institution, and all participants provided written informed consent. The investigation conformed to the principles outlined in the Declaration of Helsinki [11].

### Study design, population, and data collection

This single-center prospective cohort study was conducted at our institution. In this study, patients who do not meet the following criteria beforehand were enrolled: patients who required emergency revascularization for conditions such as acute myocardial infarction and unstable angina, those with atrial fibrillation or frequent premature ventricular contractions, those with sinus tachycardia that does not improve with β-blockers, those with implanted pacemakers, those with claustrophobia, and those who were unable to maintain a quiet recumbent position. We enrolled 71 culprit lesions from 61 patients with stable coronary artery disease who had significant coronary artery stenosis identified using invasive coronary angiography and were evaluated using CMR with non-contrast T1-weighted imaging before revascularization between October 2020 and August 2021. The exclusion criteria were as follows: (1) patients for whom adequate analysis of CMR findings could not be obtained; (2) those who did not undergo PCI; and (3) those for whom NIRS-IVUS was not used during PCI. Demographic data, coronary risk factors, and medication information were obtained from the institutional database. Blood samples were collected before the procedure.

### CMR imaging acquisition and analysis

Coronary plaque imaging was performed using a 3 T CMR scanner (MAGNETOM Skyra; Siemens Healthineers, Erlangen, Germany) with 18-channel body matrix coils. This study was performed according to conventional protocols by developing a highly efficient magnetic resonance imaging method for coronary artery plaque characterization: CATCH using an integrated anatomical reference technique [8]. CATCH is not a commercial sequence program. The CATCH technique is designed to provide the following. After scout imaging to localize the heart and diaphragm, free-breathing transaxial cine images were acquired to determine the trigger delay time when the motion of the right coronary artery was minimal. Coronary plaque images were obtained when patients were breathing freely, with fat suppressed three-dimensional spoiled gradient-echo sequence using CATCH sequence. Inversion-recovery pulse was applied every other heartbeat, allowing interleaved acquisition of dark-blood T1-weighted images and bright-blood anatomical reference images. The dark-blood T1-weighted imaging was acquired in the first heartbeat after inversion-recovery pulse. The bright-blood anatomical reference images were acquired in the second heartbeat. The imaging parameters were as follows: repetition time/echo time, 4.9/2.5 ms; flip angle, 15°; and spatial resolution, 1.4 × 1.4 × 1.3 mm^3^.

The images were stored on a compact disc in the Digital Imaging and Communications in Medicine (DICOM) format. The data were analyzed offline using the Miele-LXIV DICOM Workstation and Viewer software (Alex Bettarini). Coronary plaque image analysis was performed by two cardiologists (T.F. and A.K.), who were blinded to the plaque information obtained using NIRS-IVUS. After confirmation of the culprit lesion on angiography, we measured the signal intensity of the coronary plaque and cardiac muscle by placing a free-hand region of interest, and calculated the plaque-to-myocardial signal intensity ratio (PMR), which was defined as the signal intensity of the coronary plaque divided by that of the cardiac muscle. As described in a previous report, a culprit lesion with a PMR ≥ 1.4 was categorized in the HIP group, whereas a culprit lesion with a PMR < 1.4 was categorized in the non-HIP group [12].

### NIRS-IVUS imaging acquisition and analysis

The culprit lesion was imaged before the intervention using NIRS-IVUS pullback. NIRS-IVUS was performed using a commercially available system (TVC Imaging System or Makoto Imaging System; Infraredx, Bedford, Massachusetts, USA). This modality combines the functions of gray-scale IVUS images and NIRS, which identifies the chemical components of coronary artery plaques as means of assessing vulnerability [13]. A NIRS-IVUS catheter was inserted distal to the culprit lesion and pulled back at a rate 0.5 mm/s, after intracoronary injection of nitroglycerin.

NIRS-IVUS analysis was accurately performed by two cardiologists (T.F. and T.D.), and the following items were measured. NIRS images quantitatively estimated the amount of lipid-rich plaque (LRP) within the culprit lesion, and were analyzed off-line. The NIRS chemogram presented data as yellow indicating the presence of LRP or red indicating the absence of LRP, and allowed calculation of the lipid core burden index (LCBI); total yellow pixels divided by total viable pixels within the region of interest multiplied by 1000 [14]. In this study, the LCBI was calculated for every 4-mm segment (LCBI_4mm_) within the culprit lesion. The maximum LCBI_4mm_ (maxLCBI_4mm_) was defined as the maximum LCBI within any 4-mm-long segment, and the lesion LCBI was defined as the total LCBI throughout the culprit lesion. A large LRP was defined as LRP with a maxLCBI_4mm_ ≥ 400, which was the cutoff value validated in previous studies [15, 16].

Gray-scale IVUS analysis was performed according to two clinical expert consensus documents [17, 18]. The proximal and distal references were the proximal and distal sites with the largest lumen within 10 mm of stenosis. The lumen and external elastic membrane (EEM) were measured by tracing the leading edge of the intima and tracing the border between the media and adventitia. The plaque plus media area was defined as the EEM area minus the lumen area. Plaque burden was calculated as the plaque plus media area divided by the EEM area multiplied by 100%. In addition, the remodeling index was calculated as the lesion site EEM cross-sectional area (CSA) divided by the average of the proximal and distal reference EEM CSA. Positive remodeling and negative remodeling were defined as a remodeling index > 1.05 and < 0.95, respectively [19]. Regarding plaque morphology, atheroma was classified as follows: fibrous plaque has an intermediate echogenicity between soft (hypoechoic) plaque and highly echogenic calcified plaques, and calcified plaque has a higher echogenicity than the adventitia with an acoustic shadow. Attenuated plaque and echo-lucent plaque were defined as previously published [20]. Attenuated plaque was identified by the absence of the ultrasound signal behind the plaque that was either hypoechoic or isoechoic to the reference adventitia, but contained no bright calcium. Echo-lucent plaque contained an intraplaque zone of absence or low echogenicity (lower than that of the reference adventitia) surrounded by tissue of greater echodensity.

### Measurement of high-sensitive cardiac troponin-T

High-sensitive cardiac troponin-T (hs-cTnT) was measured at baseline and 24 h after PCI. Hs-cTnT levels were measured using an electrochemiluminescence immunoassay (Elecsys Troponin T hs; Roche Diagnostics GmbH, Mannheim, Germany). This method has a measuring range of 0.003–10 ng/mL and a 99th percentile upper reference limit (URL) of 0.014 ng/mL with a coefficient variation of < 10% at 0.013 ng/mL.

pMI was defined as an absolute elevation in hs-cTnT values of  > 5 × 99th percentile URL in patients undergoing PCI, according to the fourth universal definition of myocardial infarction. If the baseline hs-cTnT values are increasing, then an increase of > 20% is required for the diagnosis of pMI [21].

### Statistical analysis

Categorical data are presented as number (percentage) and were compared using the chi-square test. Continuous variables are expressed as mean ± standard deviation or as median (interquartile range) and compared using one-way analysis of variance or the Kruskal–Wallis test. The Shapiro–Wilk test was used to examine whether the scores were likely to follow a certain distribution in all patients. If *p* < 0.05, a normal distribution of the variable was not considered. Univariable analysis of the relationship between the PMR and maxLCBI_4mm_ was performed using the Pearson correlation analysis. The receiver operating characteristic (ROC) curve is used to evaluate the ability of PMR to cause the disease, and this optimal cutoff point was selected by maximizing the Youden index. The discriminability of continuous variables was measured using the area under the ROC curve (AUC). Multivariable logistic regression analysis was performed to predict a binary categorical field using stepwise selection with entry/stay criteria of 0.20/0.20.

All probabilities are expressed as two-tailed values, with statistical significance set at *p* < 0.05. All confidence intervals (CIs) were computed at 95% level. All data were analyzed using JMP for Macintosh (version 14.2, SAS Institute, Cary, North Carolina, USA).

## Results

### Baseline clinical characteristics of study population

**Fig. 1:**
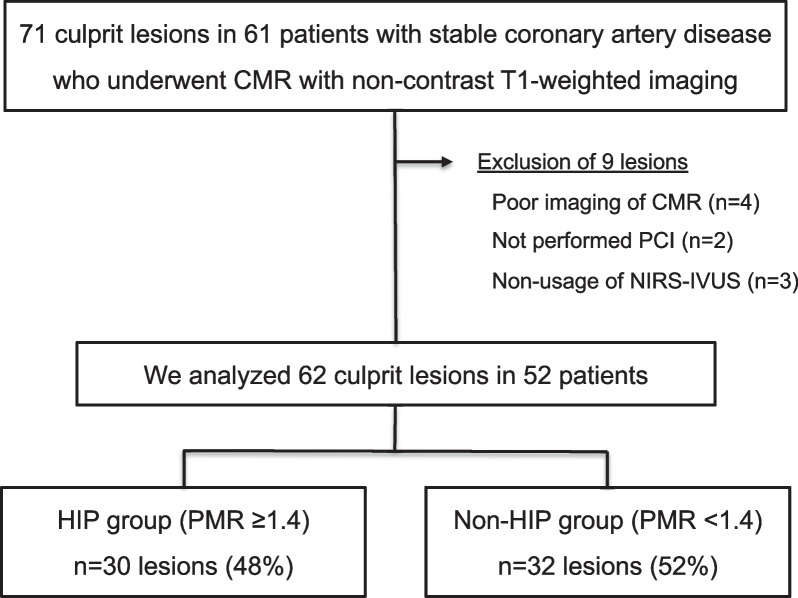
Flow chart. Among 71 culprit lesions in 61 patients with stable coronary artery disease who underwent CMR with non-contrast T1-weighted imaging, patients were excluded from this study for the following reasons: poor imaging of CMR, not performed PCI, and non-usage of NIRS-IVUS. We analyzed 62 culprit lesions in 52 patients, and these patients were divided into two groups based on the presence or absence of HIP, which were defined as PMR ≥ 1.4. 30 lesions (48%) were allocated to the HIP group, and 32 lesions (52%) were allocated to the non-HIP group. CMR, cardiovascular magnetic resonance; HIP, high-intensity plaque; IVUS, intravascular ultrasound; NIRS, near infrared spectroscopy; PCI, percutaneous coronary intervention; PMR, plaque-to-myocardial signal intensity ratio

Overall, we studied 62 culprit lesions in 52 patients with stable coronary artery disease who underwent CMR with non-contrast T1-weighted imaging and PCI using NIRS-IVUS. We excluded four lesions for which adequate analysis of CMR findings could not be obtained, two lesions for which PCI was not performed, and three lesions for which NIRS-IVUS was not used during PCI. Based on a PMR ≥ 1.4, 30 lesions (48%) were allocated to the HIP group, and 32 lesions (52%) were allocated to the non-HIP group (Fig. 1).

The baseline clinical characteristics of the patients are summarized in Table 1. Patients’ mean age was 67 ± 9 years, and 81% were men. The prevalence of hypertension, diabetes mellitus, current smoking, family history of premature coronary artery disease, and chronic kidney disease was 90%, 42%, 19%, 17%, and 21%, respectively. In addition, all 52 patients were taking statins, and half of them were taking ezetimibe. This study provided adequate lipid control, as shown by the median low-density lipoprotein cholesterol value of 68 mg/dL.

**Table 1:** Patient clinical characteristics

	Overall 52 patients
*Baseline characteristics*	
Age, years	67 ± 9
Male, n (%)	42 (81)
Body mass index, kg/m^2^	23.5 (22.3–26.2)
Hypertension, n (%)	47 (90)
Diabetes mellitus, n (%)	22 (42)
Current smoker, n (%)	10 (19)
Family history of premature CAD, n (%)	9 (17)
Chronic kidney disease, n (%)	11 (21)
*Laboratory data*	
LDL-C, mg/dL	68 (56–89)
HDL-C, mg/dL	45 (37–56)
LDL-C to HDL-C ratio	1.6 (1.1–2.2)
Triglyceride, mg/dL	107 (80–155)
Hemoglobin A1c, %	6.0 (5.7–6.6)
Estimated GFR, mL/min/1.73 m^2^	69 (62–86)
Pre-procedural hs-cTnT, ng/mL	0.010 (0.007–0.019)
*Medications*	
Aspirin use, n (%)	52 (100)
P2Y_12_ receptor inhibitor use, n (%)	52 (100)
β-blocker use, n (%)	31 (60)
ACE-i / ARB use, n (%)	26 (50)
Statin use, n (%)	52 (100)
Ezetimibe, n (%)	26 (50)

ACE-i, angiotensin-converting enzyme inhibitor; ARB, angiotensin receptor blocker; CAD, coronary artery disease; GFR, glomerular filtration rate; HDL-C, high-density lipoprotein cholesterol; hs-cTnT, high-sensitivity cardiac troponin T; LDL-C, low-density lipoprotein cholesterol

### Procedural and imaging characteristics

Table 2 shows the procedural and imaging characteristics of the lesions with and without HIP. In the CMR analysis, the median PMR was 1.37 (interquartile range [IQR], 1.13–1.58), and the HIP group had a significantly higher PMR than the non-HIP group (1.61 [IQR, 1.43–2.11] versus [vs.] 1.14 [IQR, 1.00–1.26], *p* < 0.001).There was no significant difference in left ventricular (LV) ejection fraction and LV mass between the two groups.

**Table 2:** Lesion iImaging characteristics

	Overall n = 62	HIP group n = 30	Non-HIP group n = 32	*p* value
*Lesion and procedure*				
Left anterior descending artery, n (%)	35 (56)	15 (50)	20 (63)	0.321
Right coronary artery, n (%)	13 (21)	6 (20)	7 (22)	0.856
Left circumflex artery, n (%)	14 (23)	9 (30)	5 (16)	0.174
*CMR findings*				
Plaque-to-myocardial signal intensity ratio	1.37 (1.13–1.58)	1.61 (1.43–2.11)	1.14 (1.00–1.26)	< 0.001
Left ventricular ejection fraction, %	61.6 (54.0–67.4)	63.0 (54.0–66.3)	58.8 (53.9–68.6)	0.746
Left ventricular mass, g	71.1 (59.6–90.8)	74.6 (63.8–99.8)	68.9 (56.8–88.3)	0.335
*NIRS findings*				
MaxLCBI_4mm_	391 ± 222	500 ± 170	288 ± 218	< 0.001
MaxLCBI_4mm_ ≥ 400, n (%)	26 (42)	19 (63)	7 (22)	< 0.001
Lesion LCBI	132 ± 83	170 ± 76	97 ± 74	< 0.001
*IVUS findings*				
Pull-back length, mm	61.3 ± 24.2	68.5 ± 25.2	54.5 ± 21.5	0.021
Lesion length, mm	20.7 (13.8–33.5)	22.8 (13.4–35.9)	19.1 (14.6–32.6)	0.473
*Plaque morphology*				
Fibrous plaque, n (%)	28 (45)	9 (30)	19 (59)	0.019
Attenuated plaque, n (%)	25 (40)	15 (50)	10 (31)	0.132
Echo-lucent plaque, n (%)	13 (21)	10 (33)	3 (9)	0.018
Calcified plaque, n (%)	34 (55)	17 (57)	17 (53)	0.779
Arc of calcified plaque within maxLCBI_4mm_, °	23 (0–84)	25 (0–100)	23 (0–70)	0.589
Remodeling index	1.00 ± 0.10	1.03 ± 0.07	0.96 ± 0.11	0.009
Average reference EEM CSA, mm^2^	11.6 (9.6–14.7)	10.8 (9.0–13.3)	12.4 (10.2–16.6)	0.078
EEM CSA within maxLCBI_4mm_, mm^2^	11.9 (9.3–13.7)	10.9 (9.0–13.2)	12.0 (9.5–15.7)	0.426
Lumen CSA within maxLCBI_4mm_, mm^2^	3.7 (2.9–5.1)	3.2 (2.6–4.0)	4.3 (3.6–5.7)	0.004
Plaque burden within maxLCBI_4mm_, %	66 ± 10	69 ± 8	63 ± 10	0.011

HIP group, patients with PMR ≥ 1.4 (n = 30)

Non-HIP group, patients with PMR < 1.4 (n = 32)

CMR, cardiovascular magnetic resonance; CSA, cross-sectional area; EEM, external elastic membrane; HIP, high-intensity plaque; IVUS, intravascular ultrasound; LCBI, lipid-core burden index; maxLCBI_4mm_, maximum lipid core burden index calculated for every 4-mm segment; NIRS, near infrared spectroscopy

The NIRS-IVUS measures for the corresponding lesions are shown in Table 2. The mean and median maxLCBI_4mm_ were 391 ± 222 and 388 (IQR, 229–558), respectively; the mean and median lesion LCBI were 132 ± 83 and 122 (IQR, 70–203), respectively; and the presence of large LRP accounted for 42%. Regarding plaque morphology, fibrous, attenuated echo-lucent, and calcified plaques were observed in 28 (45%), 25 (41%), 13 (21%), and 34 (55%) lesions, respectively. The median arc of calcified plaque within the maxLCBI_4mm_ was 23° (range 0–84°).

**Fig. 2:**
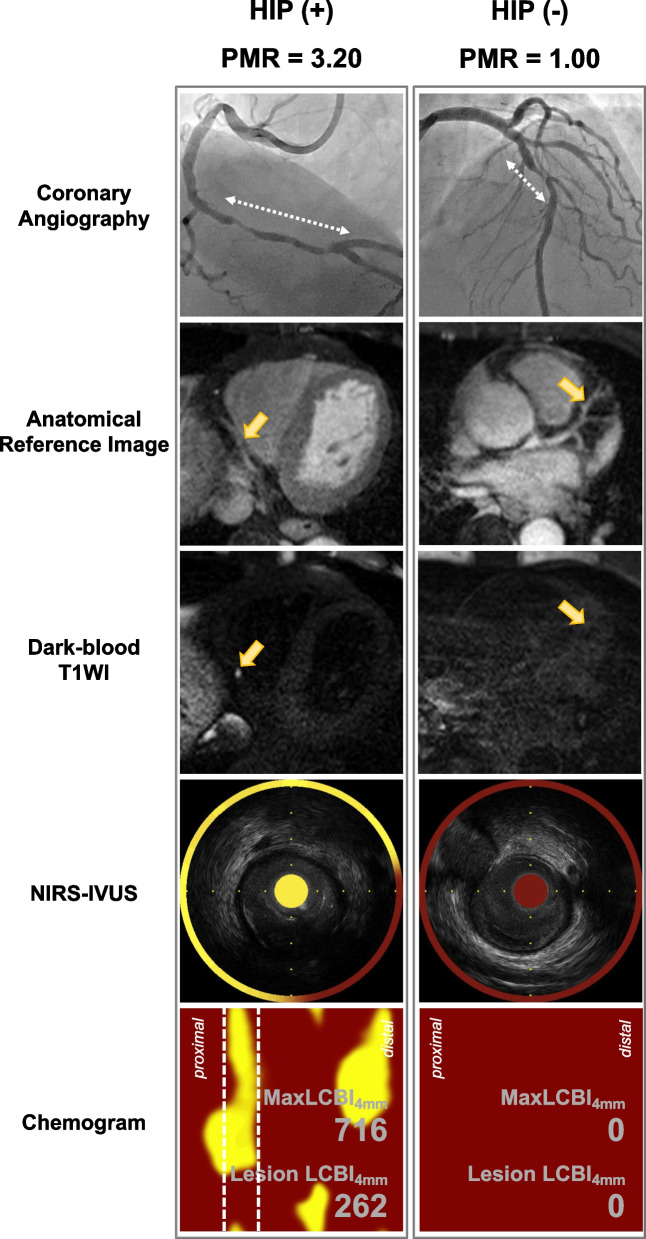
Representative images of coronary plaque with and without high intensity plaque (HIP). The left panel shows the images of a patient with HIP, and the right panel shows the images of a patient without HIP. A culprit lesion site is detected on coronary angiography (white dotted arrow). After identifying the same site using an anatomical reference image, the presence of HIP was confirmed using dark-blood T1-weighted imaging (yellow arrow). In addition, NIRS-IVUS showed plaque morphology (soft plaque or fibrous plaque) and the maximum LCBI at the 4-mm segment using a chemogram. LCBI, lipid-core burden index; NIRS, near infrared spectroscopy

The HIP group had a significantly higher maxLCBI_4mm_, remodeling index, plaque burden within the maxLCBI_4mm_, and proportion of echo-lucent plaque than the non-HIP group (all, *p* < 0.05). Representative images of the coronary plaques with HIP and without HIP are shown in Fig. 2.

### Relationships between max LCBI_4mm_ and PMR

**Fig. 3:**
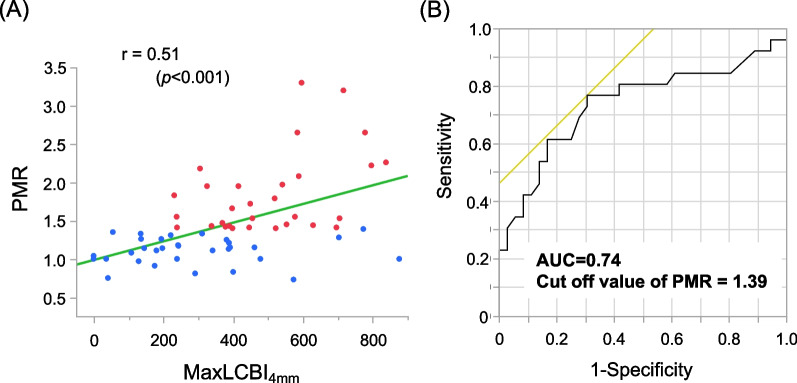
Relationship between PMR and maxLCBI_4mm_. **A** Correlation analysis of the PMR and maxLCBI_4mm_. **B** ROC curve analysis for predicting a maxLCBI_4mm_ ≥ 400. AUC, area under the receiver operating characteristic curve; maxLCBI_4mm_, maximum 4-mm lipid-core burden index; LCBI, lipid-core burden index; PMR, plaque-to-myocardial signal intensity ratio; ROC, receiver operating characteristic

The correlation between the maxLCBI_4mm_ and PMR was significantly positive, as shown in Fig. 3A (r = 0.51, *p* < 0.001). In addition, the AUC was computed to test the predictive discrimination of the maxLCBI_4mm_ ≥ 400. For a PMR cutoff value of 1.39, the AUC was 0.74 (Fig. 3B); this had the highest discriminating sensitivity (0.77) and specificity (0.69).

In multivariable logistic regression analysis, the NIRS-derived large LRP (odds ratio [OR] = 5.41; 95% CIs 1.65–17.8, *p* = 0.005) and IVUS-derived echo-lucent plaque (OR = 5.12; 95% CIs 1.11–23.6, *p* = 0.036) were the strong independent predictors of HIP (Table 3).

**Table 3:** Univariable and multivariable logistic regression analysis for prediction of HIP (PMR ≥ 1.4)

Variable	Univariable		Multivariable	
	OR (95% CIs)	*p* value	OR (95% CIs)	*p* value
NIRS-derived large LRP (maxLCBI_4mm_ ≥ 400)	6.17 (2.01–18.9)	< 0.001	5.41 (1.65–17.8)	0.005
IVUS-derived echo-lucent plaque	4.83 (1.18–19.8)	0.018	5.12 (1.11–23.6)	0.036
IVUS-derived plaque burden of ≥ 70%	2.24 (0.78–6.41)	0.130	1.89 (0.57–6.33)	0.300
IVUS-derived attenuated plaque	2.20 (0.78–6.19)	0.132		

CIs, confidence intervals; HIP, high-intensity plaque; IVUS, intravascular ultrasound; LRP, lipid-rich plaque; NIRS, near-infrared spectroscopy; maxLCBI_4mm_, maximum lipid core burden index calculated for every 4-mm segment; OR, odds ratio; PMR, plaque-to-myocardial signal intensity ratio

### Association between pMI and presence of HIP

**Fig. 4:**
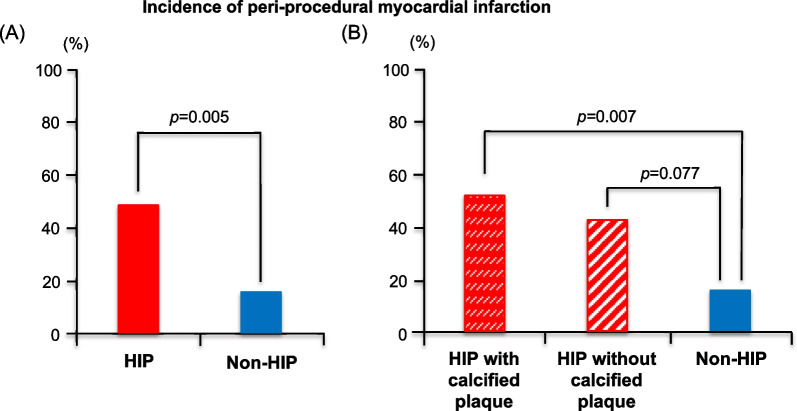
Incidence of peri-procedural myocardial infarction. **A** Comparison between the HIP and non-HIP groups. **B** Comparison between the HIP subgroups according to the presence of calcified plaque and the non-HIP group. HIP, high-intensity plaque

Of the 61 cases, 19 had an elevated baseline hs-cTnT level (> 99th percentile URL): 13 of these 19 cases had a post-procedural hs-cTnT level > 5 × 99th percentile URL, and 19 cases finally met the defined criteria and were categorized as having pMI. Overall, pMI occurred in 14 of 30 lesions (47%) with HIP, compared to 5 of 32 lesions (16%) without HIP (*p* = 0.005), as shown in Fig. 4A.

The patients with pMI had a significantly higher PMR value (1.47 [IQR, 1.15–2.18] vs. 1.27 [IQR, 1.07–1.43], *p* = 0.014), and higher rate of the presence of HIP (74% vs. 36%, *p* = 0.005). However, there was no significant difference in the maxLCBI_4mm_ value and proportion of large LRP between the cases with and without pMI. In addition, the AUC was computed to test the predictive discrimination of pMI. For a PMR cutoff value of 1.42, the AUC was 0.70, which had the highest discriminating sensitivity (0.68) and specificity (0.74).

In multivariable logistic regression analysis for the prediction of pMI incidence, the CMR-derived HIP (OR = 5.68; 95% CIs 1.53–21.1, *p* = 0.009) and chronic kidney disease (OR = 6.24; 95% CIs 1.46–26.6, *p* = 0.013) were independent predictors, but not NIRS-derived large LRP and IVUS-derived echo-lucent plaque (Table 4).

**Table 4:** Univariable and multivariable logistic regression analysis for prediction of incident peri-procedural myocardial infarction

Variable	Univariable		Multivariable	
	OR (95% CIs)	*p* value	OR (95% CIs)	*p* value
CMR-derived HIP (PMR ≥ 1.4)	5.04 (1.52–16.7)	0.005	5.68 (1.53–21.1)	0.009
Chronic kidney disease	5.38 (1.46–19.8)	0.010	6.24 (1.46–26.6)	0.013
NIRS-derived large LRP (maxLCBI_4mm_ ≥ 400)	2.00 (0.67–6.01)	0.215		
IVUS-derived echo-lucent plaque	0.98 (0.26–3.68)	0.974		

CIs, confidence intervals; CMR, cardiovascular magnetic resonance; HIP, high-intensity plaque; IVUS, intravascular ultrasound; LRP, lipid-rich plaque; NIRS, near-infrared spectroscopy; maxLCBI_4mm_, maximum lipid core burden index calculated for every 4-mm segment; OR, odds ratio; PMR, plaque-to-myocardial signal intensity ratio

## Discussion

The major findings of our study include: (1) In the culprit plaque analysis, a significant positive correlation was found between the PMR measured by CMR and the maxLCBI_4mm_ on NIRS; (2) multivariable logistic regression analysis revealed that NIRS-derived large LRP and IVUS-derived echo-lucent plaque were the strong independent predictors of HIP; (3) the incidence of pMI was significantly associated with the presence of HIP, but not with the presence of large LRP and echo-lucent plaque; and (4) the AUC of pMI was 0.70 for a PMR cutoff value of 1.42, so the PMR could be a useful predictor of the pMI incidence.

We confirmed that the CATCH technique was a feasible CMR method as a routine clinical protocol, because this study revealed that the presence of CMR-derived HIP was a strong independent predictor for NIRS-derived large LRP, and high predictive ability was shown in ROC curves analysis. However, a recent study on the association between the presence of HIP detected by the CATCH technique using CMR and the imaging findings of NIRS-IVUS reported that the presence of HIP was significantly associated with the intraplaque hemorrhage (IPH) features detected on IVUS, but not with the characteristic lipid-pool findings on NIRS [22].

Liu et al. demonstrated that HIPs on T1-weighted imaging by CMR represented histopathological IPH in the carotid plaques, although the relationship with coronary HIPs was not clarified because the frequency of IPH was quite low in coronary arteries [23]. A recent study on the histopathological characterization of HIP in coronary plaques was reported. Uzu et al. showed that HIP lesions had significantly higher not only IPH but also atheroma plaque [24]. Additionally, the patients enrolled in the present study had a higher prevalence of hypertension and diabetes mellitus compared to a study by Sato et al. Thus, we assumed that the present study had a higher proportion of large LRP due to more vulnerable plaques, although the correlation between the maxLCBI_4mm_ and PMR was almost similar in both studies. Some studies reported the relationship between histopathological IPH and NIRS-IVUS findings. Matsumura et al. showed that the features suggestive of IPH on NIRS-IVUS could be a greater IVUS-derived plaque burden, a higher NIRS-derived LCBI, and an IVUS-derived echo-lucent plaque [25]. In addition, Pu et al. reported that segments with echo-lucent plaque contained not only the histological IPH but also the necrotic cores or lipid pools, and 60% of echo-lucent plaque contained NIRS-derived large LRP [26]. Furthermore, the necrotic cores are formed from macrophage infiltration of lipid pools and cell death, followed by secondary necrosis due to cholesterol clefts, microcalcification, and IPH. IPH appears to be a process in the progression of necrotic cores [27]. The present study showed that NIRS-derived large LRP and IVUS-derived echo-lucent plaque were independently predictors for the presence of HIP as shown in Table 3, although these were not in a significant causal relationship (Additional file 1: Table S1). Thus, result of the present study would be reasonable after considering that CMR-derived HIP, NIRS-derive large LRP, and IVUS-derived echo-lucent plaque each have reflected lipid-rich necrotic core and IPH. The accumulation of research data on the relationship between HIP and NIRS-IVUS findings in autopsy cases would lead to clarification.

Approximately one-third of all elective PCI procedures are associated with an incidence of pMI, which is related to increased subsequent mortality [28]. Stone et al. reported that pMI developed more in the LRP group than in the non-LRP group, using a maxLCBI_4mm_ cutoff value ≥ 600 to define LRP [7]. We confirmed that pMI developed in 31% of cases; pre-procedural hs-cTnT levels, prevalence of cerebrovascular disease and renal failure, and proportion of attenuated plaque and HIP were significantly higher in cases with pMI than in cases without pMI in our study. However, there was no significant difference in the maxLCBI_4mm_ value and presence of large LRP between both groups. As a result, the presence of CMR-derived HIP was a significantly strong predictor for the incidence of pMI, and a PMR cutoff value of 1.42 was found to be the optimal cutoff point for prediction. The data in our study are consistent with previously published data on the association between the presence of HIP and incidence of pMI [10, 29]. We believe that coronary plaque detection using the CATCH technique is a valuable non-invasive modality to assess the risk of myocardial damage without invasive and direct observation, and use of contrast agents.

**Fig. 5:**
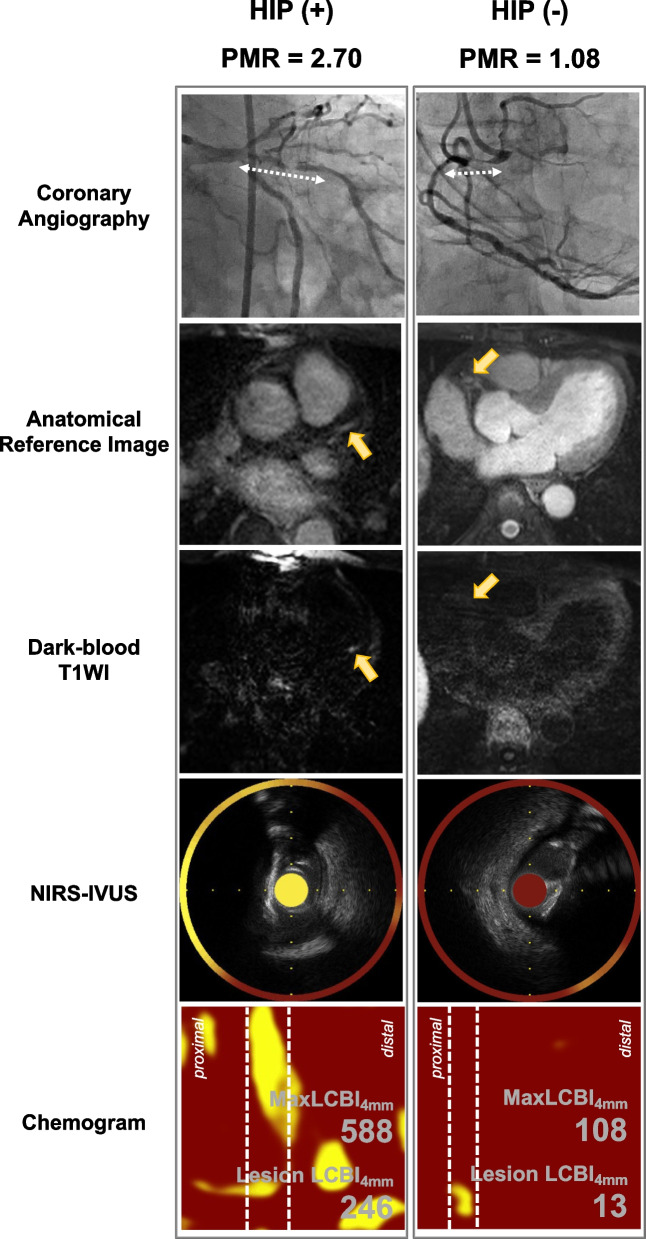
Representative images of coronary culprit lesions with calcified plaque. The left panel shows the images of a patient with HIP and calcified plaque, and the right panel shows the images of a patient with non-HIP and calcified plaque. A culprit lesion site is detected on coronary angiography (white dotted arrow). After identifying the same site using an anatomical reference image, the presence of HIP is confirmed using dark-blood T1-weighted imaging (yellow arrow). In addition, NIRS-IVUS shows a calcified plaque and maximum LCBI at the 4-mm segment using a chemogram. T1w, T1 weighted image

Herrmann et al. reported that calcified plaques are related to increased post-procedural hs-cTnT levels and mortality [30]. Thus, we focused on the relationship between the presence of calcified plaques and HIPs and the incidence of pMI; we confirmed that the HIP subgroup with calcified plaque had a significantly higher rate than the non-HIP group (53% vs. 16%, *p* = 0.007; Fig. 4B). Representative images of coronary plaques in the HIP and non-HIP groups with calcified plaques are shown in Fig. 5. Calcified plaques appear hypointense on T1-weighted images but not on ultrashort echo time images [31], thus, coronary plaque detection using CMR may contribute to predicting cardiovascular events more sensitively. Especially, the risk assessment may be promising in patients with renal dysfunction who have difficulty using contrast or in patients with diabetes mellitus who are characterized by coronary artery calcification.

## Limitations

Our study has several limitations. First, as this was a single-center prospective cohort study, unknown confounding factors might have affected the outcomes, regardless of analytical adjustments, and the small number of enrolled patients limited the statistical power of the study. Second, the spatial resolution is one of the difficulties in CMR coronary artery plaque imaging. The value of PMR may be affected by the partial volume, motion, R-R interval variation, magnetic field inhomogeneity, and instability of diaphragm synchrony. Thus, we tried to stabilize the R-R interval by controlling the pulse rate and use the optimal inversion time and flip angle, to minimize the change in PMR. In addition, this technique determines the trigger delay time when the motion of the right coronary artery was minimal, thus, the distal delineation of left anterior descending artery and left circumflex artery could be relatively obscured. Therefore, the variability and reliability of PMR also require further investigation. Third, the lack of standardization and quantitative nature of diagnosing HIPs by CMR, which is based on ‘eye-ball’ nature of this diagnosis and manual regions of interest tracings, could be one of the limitations in this research. In addition, we mentioned that the PMR cutoff value ≥ 1.4 defined HIP, which is frequently used in a single-center prospective study. However, the PMR cutoff value of 1.0 has also been used in other studies [9, 32]. Thus, the best cutoff PMR threshold of HIP for risk stratification is still not clear. Fourth, the non-contrast T1-weighted imaging by CMR, which is used in the present study is limited in plaque burden and degree of stenosis. CATCH method is designed to largely suppress tissue types with normal range of T1-weighted imaging and facilitate the identification of HIPs among mostly dark background tissues. However, it also became difficult to identify plaques that were not of high intensity; therefore we could not reliably assess the plaque burden. In addition, the reference imaging is sufficient for anatomical localization but may not have the ideal image contrast as a dedicated CMR angiography.

## Conclusions

There is an important relationship between coronary HIPs on non-contrast T1-weighted imaging measured by CMR and vulnerable plaque features measured by NIRS-IVUS. As a result, coronary plaque assessment by CMR could be useful in differentiating the high-risk lesions for pMI from low-risk lesions, and that a threshold PMR ≥ 1.4 is clinically suitable. Thus, we believe that non-contrast T1-weighted imaging in CMR using the CATCH technique can be useful not only for characterization of vulnerable plaque features, but also for pre-PCI risk stratification.

## Supplementary Information

**Additional file 1: Table S1.** Univariable and multivariable logistic regression analysis for prediction of large LRP (maxLCBI_4mm_ ≥ 400).

## Data Availability

The datasets during and/or analyzed during the current study are available from the corresponding author with reasonable request.

## Abbreviations

AUC: Area under the receiver operating characteristic curve
CATCH: Coronary atherosclerosis T1-weighted characterization
CIs: Confidence intervals
CMR: Cardiovascular magnetic resonance
CSA: Cross-sectional area
DICOM: Digital Imaging and Communications in Medicine
EEM: External elastic membrane
HIPs: High-intensity plaques
hs-cTnT: High-sensitive cardiac troponin T
IPH: Intraplaque hemorrhage
IQR: Interquartile range
IVUS: Intravascular ultrasound
LCBI: Lipid core burden index
LCBI_4mm_: Lipid core burden index calculated for every 4-mm segment
LRP: Lipid-rich plaque
LV: Left ventricle/left ventricular
maxLCBI_4mm_: Maximum lipid core burden index calculated for every 4-mm segment
NIRS: Near-infrared spectroscopy
NIRS-IVUS: Near-infrared spectroscopy and intravascular ultrasound
OR: Odds ratio
PCI: Percutaneous coronary intervention
pMI: Peri-procedural myocardial infarction
PMR: Plaque-to-myocardial signal intensity ratio
ROC: Receiver operating characteristic
URL: Upper reference limit
vs.: Versus
