# Supplementary material for: Relationship between coronary high-intensity plaques on T1-weighted imaging by cardiovascular magnetic resonance and vulnerable plaque features by near-infrared spectroscopy and intravascular ultrasound: a prospective cohort study
Source: J Cardiovasc Magn Reson. 2023 Jan 30;25:4. doi: 10.1186/s12968-023-00916-1 (PMC9885661; doi:10.1186/s12968-023-00916-1)
Supplement: Supplementary file 1 — Additional file 1: Table S1. Univariable and multivariable logistic regression analysis for prediction of large LRP (maxLCBI4mm ≥ 400). [file 12968_2023_916_MOESM1_ESM.docx]

| Variable | Univariable | |  | Multivariable | |
| --- | --- | --- | --- | --- | --- |
|  | OR (95% CIs) | *p* value |  | OR (95% CIs) | *p* value |
| CMR-derived HIP (PMR ≥1.4) | 6.17 (2.01-18.9) | <0.001 |  | 5.25 (1.58-17.4) | 0.007 |
| IVUS-derived attenuated plaque | 4.80 (1.61-14.3) | 0.005 |  | 4.29 (1.28-14.4) | 0.018 |
| IVUS-derived plaque burden of ≥70 % | 2.60 (0.90-7.50) | 0.074 |  | 2.00 (0.58-6.88) | 0.273 |
| IVUS-derived echo-lucent plaque | 1.84 (0.54-6.32) | 0.330 |  |  |  |

Table S1. Univariable and multivariable logistic regression analysis for prediction of large LRP (maxLCBI_4mm_ ≥400)

CIs, confidence intervals; CMR, cardiovascular magnetic resonance; HIP, high-intensity plaque; IVUS, intravascular ultrasound; LRP, lipid-rich plaque; maxLCBI_4mm_, maximum lipid core burden index calculated for every 4-mm segment; OR, odds ratio; PMR, plaque-to-myocardial signal intensity ratio.
